# Microbiological Characterization of Actinotignum schaalii Strains Causing Invasive Infections during a Multiyear Period in a Large Canadian Health Care Region

**DOI:** 10.1128/spectrum.03442-22

**Published:** 2022-11-21

**Authors:** Anthony Lieu, Jordan Mah, Gisele Peirano, Ranjani Somayaji, Deirdre Church

**Affiliations:** a Section of Infectious Diseases, Department of Medicine, University of Calgary, Calgary, Alberta, Canada; b Department of Pathology and Laboratory Medicine, University of Calgary, Calgary, Alberta, Canada; University of Texas Southwestern Medical Center

**Keywords:** *Actinotignum schaalii*, *Actinobaculum schaalii*, MALDI-TOF MS, 16S rRNA gene sequencing, phylogenetic analysis, uropathogen, emerging infection, urine culture, anaerobic infection, bloodstream infection, skin and soft tissue infection

## Abstract

Actinotignum schaalii is an underrecognized Gram-positive bacillus that is associated with urinary tract infections and cutaneous abscesses. The role of A. schaalii in invasive infections continues to be unappreciated because the bacteria can be isolated from a diverse spectrum of clinical specimens, ranging from being a single pathogen in urine and blood cultures to being deemed a colonizer in polymicrobial anaerobic cultures of sterile fluids and tissues. We conducted a microbiological analysis of clinical isolates obtained from 2012 through 2019. A total of 86 isolates were analyzed; 37 (43%) were from blood cultures, 35 (41%) were from deep wounds and abscesses, 6 (7%) were from urine samples, and the rest were recovered from peritoneal, kidney, and scrotal fluid samples. Urinary tract infections were clinically identified as the source of most cases of bacteremia, although no simultaneous urine cultures yielded positive results. The 16S rRNA gene sequences were available for 32 isolates (37%). Phylogenetic analysis revealed that AS.1/AS.2 strains caused a larger proportion of bloodstream infections (BSIs) (100% versus 52% [*P* = 0.01]) and trended toward a higher rate of hospitalization (91% versus 76% [*P* = 0.18]) but had a lower clindamycin MIC_90_ (0.12 versus >256 μg/mL). Our study emphasizes the emergence of A. schaalii as a pathogen in human urine samples, BSIs, and skin and soft tissue infections. It highlights the pitfalls of current laboratory methods in recovering and identifying this organism from clinical specimens, particularly urine samples. Phylogenetic analysis showed unique genotypic sequences for A. schaalii AS.1/AS.2 strains causing urosepsis, which requires further study to identify potential virulence factors.

**IMPORTANCE** Actinotignum schaalii is an underrecognized Gram-positive bacillus due to its special growth requirements and prior phenotypic identification methods, and it is often mistaken as a contaminant. It has been associated with various clinical syndromes, from urinary tract infections to cutaneous infections. The widespread use of molecular diagnostic methods allowed for improved detection. However, its role in invasive infections remains underappreciated. We conducted a detailed microbiological analysis to improve our understanding of this organism’s genotypic and phenotypic characteristics. Our results highlight the pitfalls of clinical laboratory recovery, particularly from urine cultures. Although most BSIs were caused by urinary tract infections, no simultaneous urine cultures identified A. schaalii, largely due to the failure of phenotypic methods to reliably isolate and identify this organism. Additionally, this is the first study demonstrating A. schaalii strains with differences in clinical and microbiological characteristics, raising the possibility of potential bacterial virulence factors contributing to invasive infections.

## INTRODUCTION

Actinotignum schaalii (formerly designated Actinobaculum schaalii) is an opportunistic pathogen that is emerging as an uncommon cause of invasive infections; it primarily causes urinary tract infections and cutaneous abscesses (1). Currently, the *Actinotignum* genus includes A. schaalii along with Actinotignum urinale and Actinotignum sanguinis (2). More broadly, it is within the order *Actinomycetales*, which encompasses the genera *Actinomyces*, *Arcanobacterium*, *Trueperella*, and *Actinotignum* (3).

In invasive infections, accurate organism identification of anaerobic bacteria is required to inform optimal clinical management, initiate appropriate empirical antibiotic therapy, and optimize clinical outcomes (4). A. schaalii is a fastidious bacterium that grows best under anaerobic conditions or in a 5% carbon dioxide (CO_2_) atmosphere (1). For these reasons, it is often missed by routine culture techniques, which favor aerobic conditions and shorter incubation periods, particularly for nonsterile specimens (1, 5–7). The pathogenic potential of A. schaalii alone or contributing to polymicrobial invasive infections has also been underrecognized since clinical microbiology laboratories rely on phenotypic methods for identification, dismissing A. schaalii as a coryneform bacillus, a commensal, or a contaminant (8, 9).

The widespread adoption of advanced proteomic approaches, such as matrix-assisted laser desorption ionization–time of flight mass spectrometry (MALDI-TOF MS) and genomic methods (i.e., 16S rRNA gene sequencing), have led to increased identification of this organism (6–9). However, commercial biochemical identification panels for aerobic and anaerobic Gram-positive bacilli, such as the Vitek ANC cards (bioMérieux, Marcy l’Étoile, France), and MALDI-TOF MS databases only recently included A. schaalii, in 2016 (1). Our laboratory routinely uses fast partial sequencing of the 16S rRNA gene (~500 bp of the V1 to V3 region) to identify a wide variety of Gram-positive bacilli deemed clinically relevant and isolated from sterile clinical specimens (10). Limited data on methods of identification for A. schaalii have been reported (11). Currently, there is no standardized antimicrobial susceptibility testing or reporting guidelines published for this organism; therefore, many clinical microbiology laboratories do not routinely perform antimicrobial susceptibility testing. Although disc diffusion and Etest methods are used for Gram-positive anaerobes, interpretation of results in the literature has been based on breakpoints for Streptococcus spp. or Staphylococcus spp. (1, 12, 13). We previously performed a comprehensive study of the clinical manifestations and population-based epidemiology of clinically relevant infections due to A. schaalii in our health care region (14). In this study, we conducted a detailed microbiological analysis of the previously described isolates to improve our understanding of the genotypic and phenotypic characteristics of this organism. Additionally, in constructing a phylogenetic tree using available sequencing data from a subset of clinical isolates, we aimed to determine whether distinct A. schaalii strains have clinical, phenotypic, or genotypic differences that potentially predict a unique virulence profile.

## RESULTS

### Patient demographic and clinical characteristics.

Eighty-six unique patients with A. schaalii infections were analyzed during the study period. All patients except 1 were >18 years of age. According to clinical chart review, bloodstream infections (BSIs) were predominantly caused by urinary tract infections (35/37 cases [94.6%]), while non-BSIs were primarily caused by skin and soft tissue infections (36/49 cases [73.5%]); urinary tract infections were only the cause of 10.2% (5/49 cases) of non-BSIs. Most BSIs affected patients >65 years of age (32/37 cases [86.5%]), while patients with non-BSIs were primarily <65 years of age (37/49 cases [75.5%]). Most urinary tract infections were diagnosed clinically, and isolation of A. schaalii from urine cultures was rarely seen (5/40 cases [12.5%]). Chart review identified no infectious diagnosis for 5.4% of BSIs and 12.2% of non-BSIs. Most patients (77/86 patients [90.7%]) received antibiotic therapy for a median duration of 10 days (interquartile range [IQR], 7 to 15 days).

### Microbiological characteristics.

**FIG 1:**
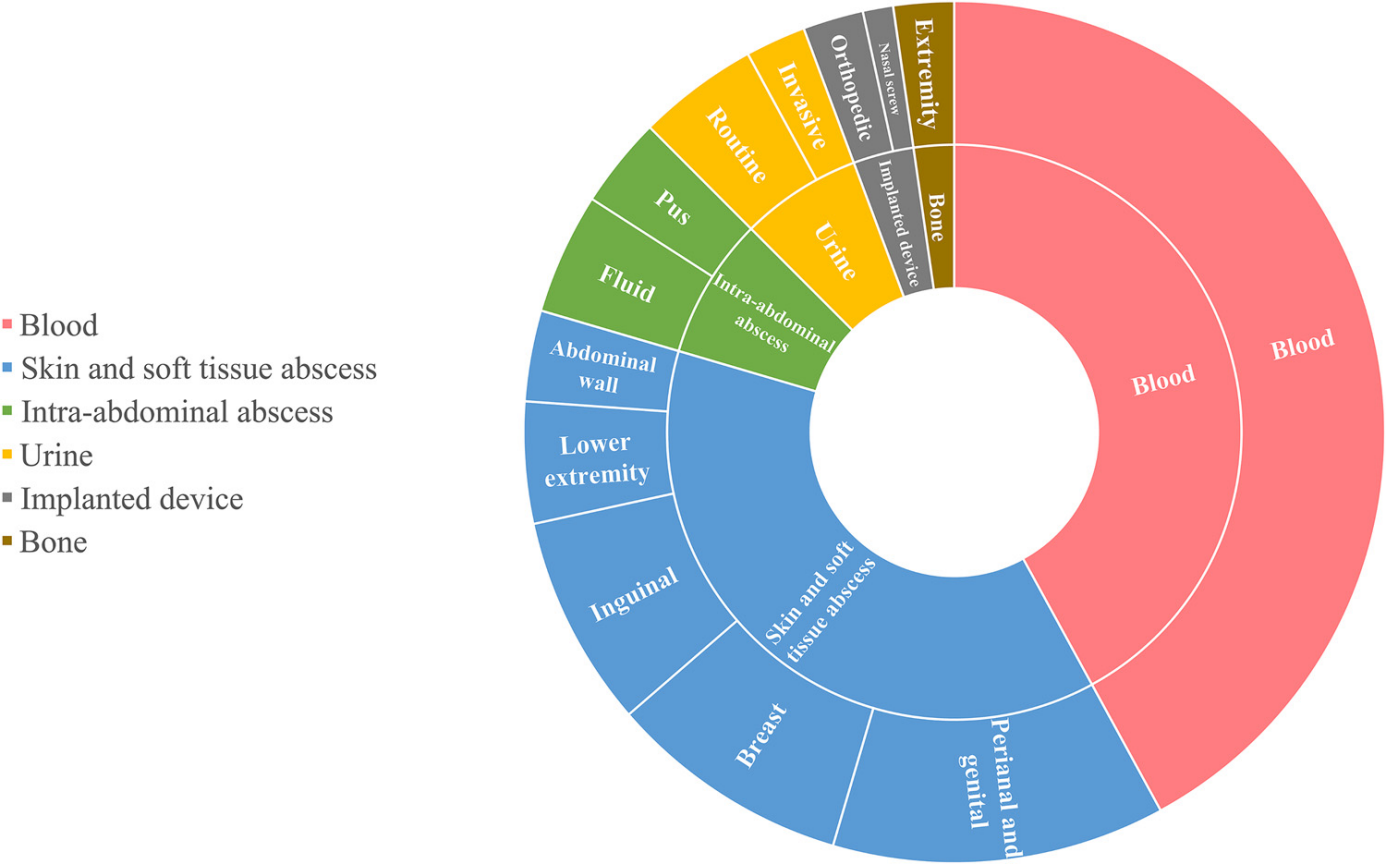
Distribution of Actinotignum schaalii clinical specimens by source. There were 86 A. schaalii isolates recovered from clinical specimens between 2012 and 2019. All samples were identified using 16S rRNA gene sequencing or MALDI-TOF MS. The sunburst chart is color-coded according to the clinical specimen source; pink represents blood specimens, blue represents skin and soft tissue abscess specimens, green represents intra-abdominal abscess specimens, yellow represents urine samples, gray represents implanted device specimens, and brown represents bone specimens.

The distribution of A. schaalii in all clinical specimen sources is outlined in Fig. 1, with a total of 86 unique isolates identified. Most invasive A. schaalii infections were from blood cultures (37/86 cases [43.0%]), while the rest were isolated from a variety of other sources, i.e., 71.4% (35/49 cases) from deep wounds and abscesses, 12.2% (6/49 cases) from urine samples, of which 2 were collected by an invasive method, 6.1% (3/49 cases) from implantable devices, 4% (2/49 cases) from bone tissue, and the remaining from peritoneal, kidney, and scrotal fluid samples (1 each). Deep wound and abscess specimens were predominantly isolated from the following sources: perianal and genital, 31.4% (11/35 cases); breast, 22.9% (8/35 cases); inguinal, 20% (7/35 cases); lower extremities, 11.4% (4/35 cases).

Pure growth of A. schaalii was observed in 69.8% of all clinical specimens, and this rate decreased to 60.5% when findings were restricted to isolates without bacteria seen in Gram stains. Pure isolates were seen in larger proportions in blood specimens than in non-blood specimens (78.4% versus 63.3%), and this was more apparent when findings were restricted to specimens without other bacteria seen in Gram stains (78.4% versus 49.0%) (Table 1). Aerobic Gram-positive copathogens were commonly observed (28.0%), including skin flora (11.6%), *Enterococcus* spp. (5.5%), Staphylococcus aureus (3.5%), and Streptococcus anginosus group (3.5%), while Escherichia coli (2.3%) and Pseudomonas aeruginosa (2.3%) were the main Gram-negative bacteria observed. Anaerobic copathogens were commonly observed, being isolated from 12.7% of all specimens; in 9.3% of specimens, bacteria were seen in Gram stains but failed to grow in aerobic cultures. The latter were all observed from wound culture isolates. *Actinomyces* spp., *Prevotella* spp., and Bacteroides fragilis group were the most frequently isolated anaerobes; the rest were mixed anaerobes.

**TABLE 1:** Microbiological characteristics of Actinotignum schaalii clinical specimens

Parameter^*a*^	Data for:		
	All specimens (*n* = 86)	Blood specimens (*n* = 37 [43.0%])	Non-blood specimens (*n* = 49 [57.0%])
Pure A. schaalii growth (no. [%])	60 (69.8)	29 (78.4)	31 (63.3)
Pure A. schaalii growth without other bacteria seen on Gram stain (no. [%])	52 (60.5)	29 (78.4)	23 (49.0)
Mixed growth of ≥2 species (no. [%])	26 (30.2)	8 (21.6)	18 (36.7)
Mixed growth of ≥3 species (no. [%])	9 (10.5)	0	9 (18.4)
Copathogen (no. [%])			
Skin flora	10 (11.6)	3 (8.1)	7 (14.3)
Staphylococcus aureus	3 (3.5)	0	3 (6.1)
Staphylococcus lugdunensis	1 (1.2)	0	1 (2.0)
Streptococcus agalactiae	2 (2.3)	0	2 (4.1)
Streptococcus anginosus group	3 (3.5)	1 (1.7)	2 (4.1)
*Enterococcus* spp.	5 (5.8)	0	5 (10.2)
Escherichia coli	2 (2.3)	1 (2.7)	1 (2.0)
Pseudomonas aeruginosa	2 (2.3)	1 (1.7)	1 (2.0)
Other Gram-negative bacteria	2 (2.3)	0	2 (4.1)
*Actinomyces* spp.	3 (3.5)	0	3 (6.1)
*Prevotella* spp.	3 (3.5)	0	3 (6.1)
Bacteroides fragilis group	2 (2.3)	0	2 (4.1)
Mixed anaerobic bacteria	4 (5.8)	0	4 (8.2)
*Candida* spp.	1 (1.2)	0	1 (2.0)
Mixed bacteria that were seen in Gram stain but failed to grow in aerobic culture (no. [%])	8 (9.3)	0	8 (16.3)
Final ID with MALDI-TOF MS (no. [%])	54 (62.8)	15 (40.5)	39 (79.6)
Final ID with 16S rRNA gene sequencing (no. [%])	21 (24.4)	14 (37.8)	7 (14.3)
Final ID with both MALDI-TOF MS and 16S rRNA gene sequencing (no. [%])	11 (12.8)	8 (21.6)	3 (6.1)
Susceptibility testing performed (no. [%])	48 (55.8)	34 (91.9)	14 (28.6)
Blood culture incubation time (median [IQR]) (h)	42 (39.0–49.0)	42 (39.0–49.0)	

a ID, identification.

### Performance of MALDI-TOF MS for identification.

Prior to the MALDI-TOF MS Vitek MS v3.0 update (2016), A. schaalii was not included in the database, which resulted in 100% (*n* = 13) discordant results, compared to 16S rRNA gene sequencing, in the preceding study years. Five cases (38%) yielded no identification, and 8 (62%) discordant identifications, including Actinomyces meyeri, Actinomyces radingae, Actinomyces turicensis, *Actinomyces* spp., Clostridium tyrobutyricum, Kocuria kistrinae, Listeria grayi, Streptococcus pseudopneumoniae, and Yersinia enterolitica. Once the library was updated in 2016, MALDI-TOF MS had 100% (*n* = 11) agreement with 16S rRNA gene sequencing and became the primary method for definitive identification. During the study period, Vitek 2 ANC and GPI cards were used 11 times for identification. Before 2017, all results were discordant (*n* = 10 [100%]) with 16S rRNA gene sequencing results, including 1 result with no organism identification. Discordant identifications included Actinomyces meyeri, *Actinomyces* spp., Granulicatella adiacens, Kocuria kistrinae, Listeria grayi, Micrococcus luteus, Micrococcus lylae, and Yersinia enterolitica. Vitek 2 ANC card analysis was performed only once after 2016, with low discrimination (92%) for A. schaalii. For blood cultures, the median incubation time was 42.0 h (IQR, 39.0 to 49.0 h). Fifty-four isolates (62.8%) had definitive identification with MALDI-TOF MS and 21 (24.4%) with 16S rRNA gene sequencing; 11 isolates (12.8%) underwent identification with both methods as part of our verification (Table 1).

### Antimicrobial susceptibility.

Susceptibility testing was performed for 48 invasive isolates (55.8%), including 34 blood culture isolates (70.8%), 8 abscess and wound culture isolates (16.7%), 5 urine culture isolates (10.4%), and 1 implantable device isolate (2.1%). Antimicrobial susceptibility results for testing of ≥8 A. schaalii isolates showed 100% susceptibility to penicillin (*n* = 50), ceftriaxone (*n* = 10), meropenem (*n* = 8), and vancomycin (*n* = 22), based on the Clinical and Laboratory Standards Institute (CLSI) M45 edition 3 MIC interpretative guidelines for *Corynebacterium* spp. (Table 2) (15). For β-lactams, MIC_90_ values were universally low at <0.06 μg/mL, except for piperacillin-tazobactam (23 isolates), with MIC_90_ values of 0.25 μg/mL. Based on the CLSI M45 ED3 interpretative guidelines for *Corynebacterium* spp. (15), high rates of resistance were found for clindamycin (*n* = 13 [45%]) and trimethoprim-sulfamethoxazole (TMP-SMX) (*n* = 9 [33%]). The MIC_90_ for clindamycin was >256 μg/mL, and that for TMP-SMX was 32 μg/mL. Tetracycline, erythromycin, gentamicin, ciprofloxacin, and moxifloxacin susceptibility testing was performed for a few isolates, and results are presented in Table 2. When analyses were restricted to BSIs and non-BSIs, there were statistical differences between antibiotics. Lower resistance to clindamycin and higher resistance to TMP-SMX were observed in the BSI group, compared to the non-BSI group (20.0% versus 58.3% and 60.0% versus 0%, respectively).

**TABLE 2:** Antimicrobial susceptibility testing and MIC distribution for A. schaalii based on CLSI and EUCAST v12.0 breakpoints for *Corynebacterium* spp.

Antimicrobial agent	No. of isolates with MICs of:													MIC_50_ (μg/mL)	MIC_90_ (μg/mL)	Susceptible (%) using^*a*^:	
	<0.06 μg/mL	0.12 μg/mL	0.25 μg/mL	0.5 μg/mL	1 μg/mL	2 μg/mL	4 μg/mL	8 μg/mL	16 μg/mL	32 μg/mL	64 μg/mL	128 μg/mL	>256 μg/mL			CLSI breakpoint	EUCAST breakpoint
Penicillin	47	3	0	0	0	0	0	0	0	0	0	0	0	<0.06	<0.06	100	100
Piperacillin-tazobactam	16	3	2	0	2	0	0	0	0	0	0	0	0	<0.06	0.25	NA^*b*^	NA
Ceftriaxone	9	1	0	0	0	0	0	0	0	0	0	0	0	<0.06	<0.06	100	NA
Imipenem	9	0	0	0	0	0	0	0	0	0	0	0	0	<0.06	<0.06	NA	NA
Meropenem	8	0	0	0	0	0	0	0	0	0	0	0	0	<0.06	<0.06	100	NA
Metronidazole	0	0	0	0	0	0	0	0	0	0	0	0	34	>256	>256	NA	NA
Clindamycin	10	2	0	0	0	0	0	0	0	1	0	0	9	0.12	>256	55	55
TMP-SMX	5	0	1	0	0	0	0	0	0	2	1	0	0	<0.06	32	67	NA
Tetracycline	1	0	1	0	0	0	0	0	0	0	0	0	0	—^*c*^	—	—	—
Erythromycin	2	0	0	0	0	0	0	0	0	0	0	0	2	—	—	—	NA
Gentamicin	0	0	0	0	2	0	1	0	0	0	0	0	0	—	—	—	NA
Ciprofloxacin	0	0	0	0	0	1	2	0	0	0	0	0	0	—	—	—	—
Moxifloxacin	1	0	0	0	0	0	0	0	0	0	0	0	0	—	—	NA	—
Vancomycin	5	10	5	2	0	0	0	0	0	0	0	0	0	0.12	0.25	100	100

a Percentage of susceptible isolates based on CLSI and EUCAST v12.0 MIC breakpoints (15, 24).

b NA, not applicable (no established breakpoints).

c —, Antimicrobial agent was tested ≤5 times, and MIC_50_, MIC_90_, and susceptibility values were not calculated.

### Phylogenetic analysis of invasive Actinotignum schaalii strains.

**FIG 2:**
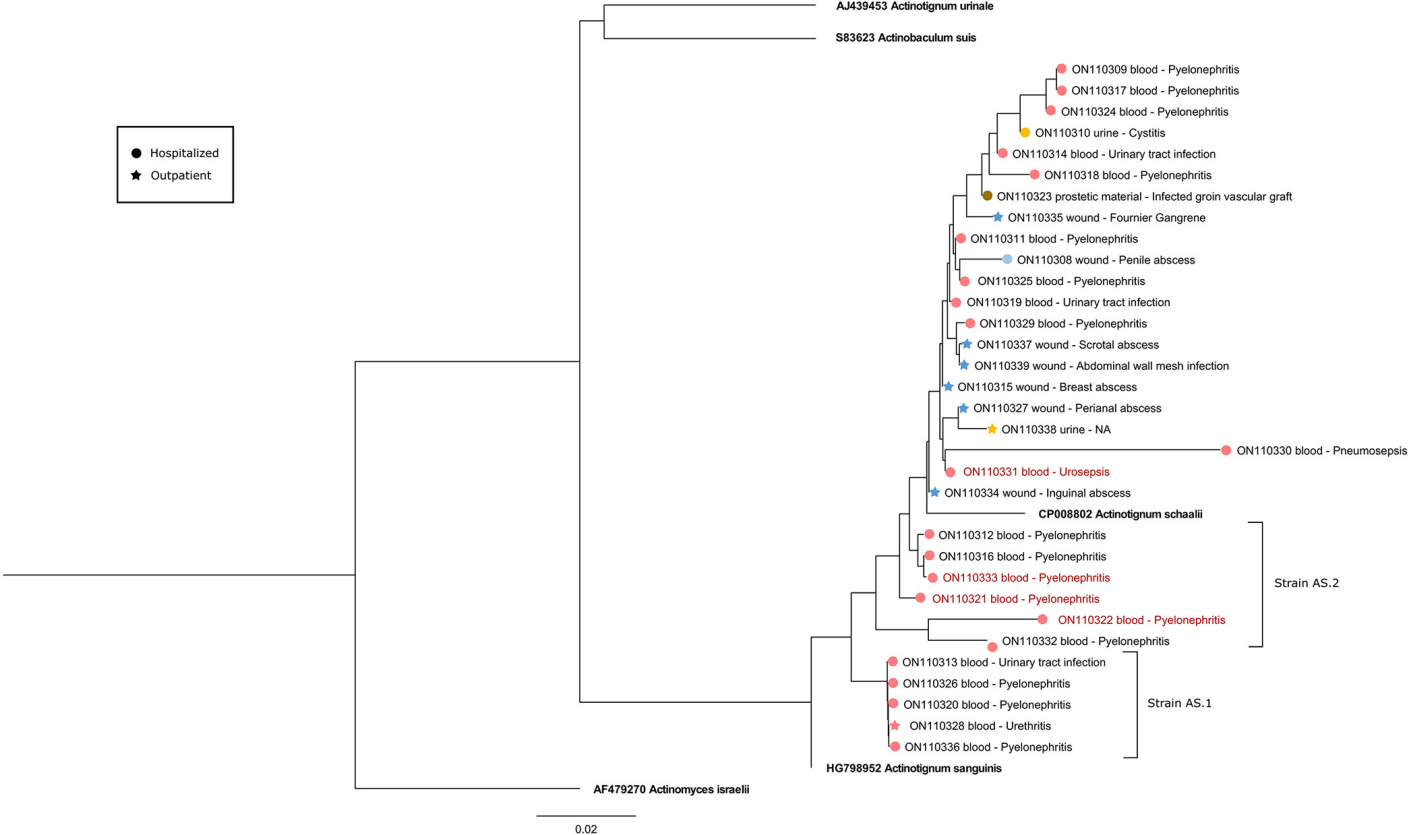
Phylogenetic tree of Actinotignum schaalii 16S rRNA gene-sequenced partial sequences. The neighbor-joining tree illustrates the phylogenetic relatedness of the sequences. The phylogenetic tree tips are labeled with the GenBank accession number and the clinical infection diagnosis. The clinical outcomes are illustrated by shape (star, outpatient; circle, inpatient) and are color-coded based on the source of infection, as in Fig. 1. The reference strains are bolded, and the red taxons represent death within 90 days. The scale bar represents the number of nucleotide substitutions per site.

Figure 2 illustrates the phylogenetic tree of all 16S rRNA gene sequences for A. schaalii strains causing BSIs and other serious invasive infections during the study period and the reference strain sequences. Our data illustrate unique strain AS.1 (GenBank accession numbers ON110313, ON110320, ON110326, ON110328, and ON110336) and unique strain AS.2 (GenBank accession numbers ON110312, ON110316, ON110321, ON110322, ON110332, and ON110333). Table 3 compares the clinical and phenotypical characteristics of strains AS.1 and AS.2 (AS.1/2) with other sequenced strains. Demographic data were similar for the two groups, but the AS.1/2 strains had a significantly larger proportion of BSIs caused by urinary tract infections, with a trend for higher hospitalization and mortality rates. This unique strain also had no detected clindamycin resistance, compared with 57.1% for the other strains.

**TABLE 3:** Clinical and phenotypic characteristics of 16S rRNA gene-sequenced A. schaalii clinical isolates by strain type

Parameter	Data for:			*P*
	All strains (*n* = 32)	AS.1/2 strains (*n* = 11)	Other strains (*n* = 21)	
Age (median [IQR]) (yr)	80.5 (54.0–85.8)	84.0 (57.0–87.0)	82.0 (62.0–86.0)	0.51
Male sex (%)	25 (78.1)	9 (81.8)	16 (76.2)	0.72
BSI (no. [%])	22 (68.8)	11 (100)	11 (52.4)	0.01
Urinary tract infection (no. [%])	23 (71.9)	11 (100)	12 (57.1)	0.02
Hospitalization (no. [%])	26 (81.3)	10 (90.9)	16 (76.2)	0.18
90-day death (no. [%])	4 (13.0)	3 (27.3)	1 (4.8)	0.08
No. with resistance to clindamycin/total no. (%)	4/15 (26.6)	0/8	4/7 (57.1)	0.12
Clindamycin MIC_50_ (μg/mL)	<0.06	<0.06	>256	
Clindamycin MIC_90_ (μg/mL)	>256	0.12	>256	

## DISCUSSION

Our unique study of A. schaalii strains recovered from a large retrospective population-based cohort of patients with invasive infections provides the largest report of isolates’ microbiological characteristics in which 16S rRNA gene-sequenced specimens were correlated with the patients’ clinical profiles (11, 16, 17). Our study highlights the previous and current pitfalls of clinical laboratory recovery and definitive identification of this important emerging pathogen. Given the Gram-positive bacillus morphology and fastidious nature of this organism, A. schaalii might have been previously overlooked as a skin contaminant or nonpathogenic coryneform bacillus in Gram stains of non-BSI specimens or analyses using available phenotypic methods, which were unable to provide an identification. Once the MALDI-TOF MS library was updated to v3.0, our internal verification showed perfect agreement (11/11 cases) with 16S rRNA gene sequencing results, and this method of identification became our laboratory’s primary routine tool for the definitive identification of Gram-positive bacilli, such as A. schaalii, recovered from the range of clinical specimens analyzed, leading to an increase in its detection. However, the number of A. schaalii strains identified in urine cultures, despite this pathogen being a primary cause of urinary tract infections, was much lower than in other studies (11, 16). This is particularly important since most of our sequences were identified in blood specimens with infections whose suspected etiology was reported to be from urinary tract infections. Similarly, all identified BSIs had no urine cultures in which A. schaalii was simultaneously identified. This highlights pitfalls in our current urine culture-based methods, which rely on chromogenic agar and nonoptimal growth conditions that do not favor the recovery of this fastidious, Gram-positive bacillus. In addition, A. schaalii appears as a white or dusty colony on Uriselect 4 chromogenic agar, produces a negative catalase reaction, and typically appears as Gram-positive bacilli on microscopic examination, leading it to be dismissed as a nonpathogenic organism.

Given the potential of this species to cause invasive BSIs, most often from ascending urinary tract infections, MALDI-TOF MS and optimal growth conditions should be considered a standard identification method for at-risk populations, especially if phenotypic characteristics suggest a Gram-positive rod. Our results showed that 86.5% of all A. schaalii BSIs involved patients >65 years of age. Therefore, assuming that BSIs came from a urinary source and would yield a positive urine culture result, use of an age cutoff of 65 years to implement routine MALDI-TOF MS analysis, with optimal growth conditions for the urine samples, 48 h of incubation, and 5% CO_2_ or anaerobic conditions, would allow for improved detection of A. schaalii in this vulnerable patient population.

A. schaalii infections were routinely identified as a constituent of polymicrobial infections, most notably in the non-BSI cases. Because A. schaalii is commonly identified as a copathogen in polymicrobial infections from deep wound and abscess cultures, this finding suggests that this organism is found as part of the normal skin and genitourinary flora. A. schaalii has been previously described as part of the microbiota of the urogenital area; however, our isolation of this organism from a variety of body sites, including breast, abdominal wall, and lower extremities, suggests that this organism may also be a colonizer of other body sites where there is a low-oxygen environment (1, 17). BSIs occurred mainly in adults >65 years of age, an age group with multiple risk factors predisposing patients to urinary tract infections and disruption of the skin barrier, which may explain why they are more prone to invasive BSIs.

There has been only one small study that compared the sequences of clinical A. schaalii isolates to compare different strains of A. schaalii using phylogenetic trees; however, the study was not correlated with clinical phenotypes (11). We demonstrated that all 11 isolates (AS.1/2) with 16S rRNA gene sequences most closely related to that of A. sanguinis and from a higher node than the reference A. schaalii isolates were all isolated from blood specimens and, according to chart review, were from a urinary tract infection, a significant finding compared to other strains. Phenotypic differences were also seen, because resistance to clindamycin was observed only for the non-AS.1/2 strains (57.1% versus 0%). In addition, from a clinical perspective, AS.1/2 strains had a greater propensity to cause BSIs, with a trend for higher hospitalizations and mortality rates, compared to the other strains. This is the first study showing potential A. schaalii strains with differences in phenotypic and clinical characteristics. Similarly, although all isolates showed low β-lactam MICs, BSI isolates had unique patterns of resistance because they tended to have lower resistance to TMP-SMX, whereas non-BSI isolates, such as isolates from abscess and wound infections, had higher resistance to clindamycin (5, 17). This finding must be interpreted with caution, given the small number of isolates tested with non-β-lactam medications. Nevertheless, this difference raises the possibility of a potential bacterial virulence factor or strains with a higher risk of BSIs (5, 16).

Although this study involved a sizeable microbiological cohort, some limitations must be considered. Given the study’s retrospective nature, the definitive identification methods were not consistently applied for all isolates. After the Vitek MALDI-TOF MS library update in 2016, MALDI-TOF MS was routinely utilized, leading to a detection bias depicted by the increase in case detection. In addition, a selection bias could have been introduced because 16S rRNA gene sequencing was performed only for an important subset of isolates causing BSIs or other types of serious life-threatening infections. Further genomic analyses are required, including whole-genome sequencing of a larger number of A. schaalii isolates to confirm the clinical relevance of the AS.1/2 strain. The total incidence rate is most likely underestimated because urine specimen isolates were not detected for the reasons outlined above.

In summary, this work further advances the understanding of A. schaalii, a slow-growing bacterium with a wide spectrum of presentation and pathogenesis. From a laboratory perspective, our study revealed that A. schaalii can still be missed by the clinical laboratory, particularly in a high-throughput laboratory with a heavy emphasis on phenotypic detection methods to rule out contaminants. In nonsterile settings, implementing MALDI-TOF MS as part of the routine diagnostic algorithm for groups at high risk for invasive A. schaalii infections would allow for better detection while limiting the increase in laboratory resources. Also, we identified strains of A. schaalii with greater propensity to cause invasive infections, suggesting genotypic factors that could influence the organism’s virulence. More extensive studies linking microbiological and clinical characteristics with outcomes are required to better understand this organism’s pathogenesis.

## MATERIALS AND METHODS

### Study design.

We completed a retrospective population-based cohort study at the centralized regional microbiology laboratory serving Calgary, Canada, and surrounding areas from 1 January 2012 to 31 December 2019. Calgary Laboratory Services (CLS) (now Alberta Precision Laboratories [APL]) is a centralized regional microbiology laboratory providing ambulatory and hospital-based testing to Calgary and the Alberta Health Services (AHS) South Zone, covering a population of 2.0 million (18). Basic demographic data for A. schaalii isolates, such as patient age and sex, were derived from the previously completed clinical study for the purposes of microbiological characterization (14). Infectious diagnosis, hospitalization, and 90-day mortality rates were determined through a retrospective chart review of the patients’ electronic medical records, which was independently performed by two investigators (J.M. and A.L.).

### Microbiological methods.

All clinical specimens with A. schaalii isolates were obtained from the laboratory information system (Millennium; Cerner, Kansas City, MO, USA) and microbiology database used by CLS Calgary Zone during the study period. Clinical specimens were processed using standard CLSI methods for blood and sterile and nonsterile fluid cultures (19). All isolates were anaerobic or aerotolerant non-spore-forming Gram-positive bacilli that were catalase negative. These key microbiological characteristics were used to confirm definitive identification. Routine urine specimens were plated on chromogenic agar (UriSelect 4; Bio-Rad, Hercules, CA), while urine samples collected through invasive methods were plated on blood agar and chromogenic agar. Chromogenic agar plates were incubated under aerobic conditions for 18 to 24 h, and blood agar plates were incubated for 48 h. Urine specimens were plated on Brucella agar and incubated for 48 h under anaerobic conditions at the physician’s request. Definitive identification was performed with the Vitek MALDI-TOF MS system (bioMérieux, Laval, Quebec) and/or 16S rRNA gene sequencing. The Vitek MS database was updated in 2016 (v3.0) to include A. schaalii. Previously, 16S rRNA gene sequencing was performed for invasive isolates only if definitive identification was required after clinical review by the microbiologist on call. Isolates were identified using a commercial MS system (Vitek MS, with ACQ software R2 v1.4.2b; bioMérieux) using MYLA v3.2.0-4 according to the manufacturer’s instructions. α-Cyano-4-hydroxycinnamic acid (CHCA) extraction was performed for all isolates using the protocol provided by the manufacturer. An aliquot of 1 μL of the extracted supernatants was placed on the steel target plate, dried, and overlaid with 1 μL of the matrix. The target plate was then loaded into the Vitek MS instrument for analysis. Samples were repeated if no identified results or low discrimination (<80% for *Enterobacteriaceae*, <90% for Streptococcus species not *Enterococcus*, and <99% for all other organisms) was obtained; 16S rRNA gene sequencing was performed when repeat MALDI-TOF MS analysis gave no identification or low discrimination. Molecular identification was performed by fast partial sequencing of the 16S rRNA gene (523 bp) with MicroSeq 500 kits and an ABI Prism 3130 sequencer (Applied Biosystems, Foster City, CA) using standard methods (10, 20, 21). A BLAST search against the SmartGene Integrated Database Network System (IDNS) for bacteria indicated the most closely related species (22). The overall identity score for all isolates was 99.9%, with 0 to 2 mismatches (22, 23). Some isolates were also tested using the Vitek ANC card (bioMérieux) but required MALDI-TOF MS or 16S rRNA gene sequencing for definitive diagnosis. Antimicrobial susceptibility testing was performed with MIC gradient strips (Etest; bioMérieux) on blood-supplemented Mueller-Hinton agar using a 0.5 McFarland standard, incubated in 5% CO_2_ at 35°C for 20 to 24 h and up to 48 h, and interpreted with CLSI M45 ED3 and European Committee on Antimicrobial Susceptibility Testing (EUCAST) v12.0 clinical breakpoints for *Corynebacterium* spp. (15, 24).

### Phylogenetic analysis.

Phylogenetic analysis of available A. schaalii isolate 16S rRNA gene sequences was conducted. The clinical strains (*n* = 32/37 strains [86.5%]) and reference sequences (*n* = 5) were aligned with MEGA v11.0.10 software (Pennsylvania State University). Four reference sequences from culture type strains were included in the phylogenetic analysis (Actinotignum schaalii [GenBank accession number CP008802], Actinotignum urinale [GenBank accession number AJ439453], Actinotignum sanguinis [GenBank accession number HG798952], and Actinobaculum suis [GenBank accession number S83623]), all of which are in the *Actinomycetales* order and *Arcanobacteriaceae* family (3). A neighbor-joining tree was inferred with 100 bootstrap replicates, and evolutionary distances were computed using the Jukes-Cantor method in MEGA (25). The tree was manually rooted on a taxonomic outlier (Actinomyces israelii [GenBank accession number AF479270]) using FigTree (26). Branches corresponding to partitions reproduced in <50% of bootstrap replicates were collapsed.

### Data analyses.

The cohort data were analyzed using standard descriptive statistics. Categorical data such as frequencies were compared using Fisher’s exact test; continuous data were summarized as medians with IQRs and compared using Wilcoxon’s rank-sum test (27). *P* values of <0.05 were considered statistically significant. SPSS v25 (IBM, Chicago, IL) was used to perform the analyses.

### Definitions.

Urinary tract infections were determined as clinician-defined urinary tract infections from the problem list in a chart review (27). Copathogens were defined as other bacteria growing in the same culture specimen as A. schaalii. Skin flora was defined as nonpathogenic commensal bacteria and included coagulase-negative Staphylococcus, viridans group streptococci, *Cutibacterium* spp., *Corynebacterium* spp., and *Propionibacterium* spp.

### Ethics approval.

A waiver of consent requirements was obtained, and the study was reviewed by the Conjoint Health Research Ethics Board (REB) and approved under certificate number REB-21-0239.

### Data availability.

All sequences were submitted to GenBank under accession numbers ON110308 to ON110339.

## References

[1] Lotte R, Lotte L, Ruimy R. 2016. *Actinotignum schaalii* (formerly *Actinobaculum schaalii*): a newly recognized pathogen—review of the literature. Clin Microbiol Infect 22:28–36. doi:10.1016/j.cmi.2015.10.038. 26577137

[2] Yassin AF, Spröer C, Pukall R, Sylvester M, Siering C, Schumann P. 2015. Dissection of the genus *Actinobaculum*: reclassification of *Actinobaculum schaalii* Lawson et al. 1997 and *Actinobaculum urinale* Hall et al. 2003 as *Actinotignum schaalii* gen. nov., comb. nov. and *Actinotignum urinale* comb. nov., description of *Actinotignum sanguinis* sp. nov. and emended descriptions of the genus *Actinobaculum* and *Actinobaculum suis*; and re-examination of the culture deposited as *Actinobaculum massiliense* CCUG 47753^T^ ( = DSM 19118^T^), revealing that it does not represent a strain of this species. Int J Syst Evol Microbiol 65:615–624. doi:10.1099/ijs.0.069294-0. 25406238

[3] Salam N, Jiao JY, Zhang XT, Li WJ. 2020. Update on the classification of higher ranks in the phylum *Actinobacteria*. Int J Syst Evol Microbiol 70:1331–1355. doi:10.1099/ijsem.0.003920. 31808738

[4] Salonen JH, Eerola E, Meurman O. 1998. Clinical significance and outcome of anaerobic bacteremia. Clin Infect Dis 26:1413–1417. doi:10.1086/516355. 9636872

[5] Cattoir V. 2012. *Actinobaculum schaalii*: review of an emerging uropathogen. J Infect 64:260–267. doi:10.1016/j.jinf.2011.12.009. 22209960

[6] Pedersen H, Senneby E, Rasmussen M. 2017. Clinical and microbiological features of *Actinotignum* bacteremia: a retrospective observational study of 57 cases. Eur J Clin Microbiol Infect Dis 36:791–796. doi:10.1007/s10096-016-2862-y. 27957598 PMC5395584

[7] Horton LE, Mehta SR, Aganovic L, Fierer J. 2018. *Actinotignum schaalii* infection: a clandestine cause of sterile pyuria? Open Forum Infect Dis 5:ofy015. doi:10.1093/ofid/ofy015. 29450211 PMC5808804

[8] Stevens RP, Taylor PC. 2016. *Actinotignum* (formerly *Actinobaculum*) *schaalii*: a review of MALDI-TOF for identification of clinical isolates, and a proposed method for presumptive phenotypic identification. Pathology 48:367–371. doi:10.1016/j.pathol.2016.03.006. 27131934

[9] Prigent G, Perillaud C, Amara M, Coutard A, Blanc C, Pangon B. 2016. *Actinobaculum schaalii*: a truly emerging pathogen? New Microbes New Infect 11:8–16. doi:10.1016/j.nmni.2015.10.012. 27014462 PMC4789325

[10] Clinical and Laboratory Standards Institute. 2018. Interpretive criteria for identification of bacteria and fungi by targeted DNA sequencing, 2nd ed. CLSI guideline MM18. Clinical and Laboratory Standards Institute, Wayne, PA.

[11] Nielsen HL, Søby KM, Christensen JJ, Prag J. 2010. *Actinobaculum schaalii*: a common cause of urinary tract infection in the elderly population: bacteriological and clinical characteristics. Scand J Infect Dis 42:43–47. doi:10.3109/00365540903289662. 19883165

[12] Lotte R, Durand M, Mbeutcha A, Ambrosetti D, Pulcini C, Degand N, Loeffler J, Ruimy R, Amiel J. 2014. A rare case of histopathological bladder necrosis associated with *Actinobaculum schaalii*: the incremental value of an accurate microbiological diagnosis using 16S rDNA sequencing. Anaerobe 26:46–48. doi:10.1016/j.anaerobe.2014.01.005. 24487002

[13] Tena D, Fernández C, Lago MR, Arias M, Medina MJ, Sáez-Nieto JA. 2014. Skin and soft-tissue infections caused by *Actinobaculum schaalii*: report of two cases and literature review. Anaerobe 28:95–97. doi:10.1016/j.anaerobe.2014.05.009. 24923266

[14] Mah J, Lieu A, Somayaji R, Church D. 2022. Characterizing *Actinotignum schaalii* infections in a large Canadian healthcare region. Future Microbiol 17:1353–1362. doi:10.2217/fmb-2022-0049. 36169260

[15] Clinical and Laboratory Standards Institute. 2016. Methods for antimicrobial dilution and disk susceptibility testing of infrequently isolated or fastidious bacteria, 3rd ed. CLSI guideline M45. Clinical and Laboratory Standards Institute, Wayne, PA. 10.1086/510431 17173232

[16] Lotte L, Lotte R, Durand M, Degand N, Ambrosetti D, Michiels J-F, Amiel J, Cattoir V, Ruimy R. 2016. Infections related to *Actinotignum schaalii* (formerly *Actinobaculum schaalii*): a 3-year prospective observational study on 50 cases. Clin Microbiol Infect 22:388–390. doi:10.1016/j.cmi.2015.10.030. 26551841

[17] Olsen AB, Andersen PK, Bank S, Søby KM, Lund L, Prag J. 2013. *Actinobaculum schaalii*, a commensal of the urogenital area. BJU Int 112:394–397. doi:10.1111/j.1464-410X.2012.11739.x. 23350855

[18] Alberta Health Services. 2020. 2019–2020: report to the community, AHS map and overview. https://www.albertahealthservices.ca/assets/about/publications/ahs-ar-2020/zones.html. Accessed 14 May 2022.

[19] Clinical and Laboratory Standards Institute. 2022. Principles and procedures for blood cultures, 2nd ed. CLSI guideline M47. Clinical and Laboratory Standards Institute, Wayne, PA.

[20] Woo PCY, Lau SKP, Teng JLL, Tse H, Yuen KY. 2008. Then and now: use of 16S rDNA gene sequencing for bacterial identification and discovery of novel bacteria in clinical microbiology laboratories. Clin Microbiol Infect 14:908–934. doi:10.1111/j.1469-0691.2008.02070.x. 18828852

[21] Tamura K, Dudley J, Nei M, Kumar S. 2007. MEGA4: Molecular Evolutionary Genetics Analysis (MEGA) software version 4.0. Mol Biol Evol 24:1596–1599. doi:10.1093/molbev/msm092. 17488738

[22] Simmon KE, Croft AC, Petti CA. 2006. Application of SmartGene IDNS software to partial 16S rRNA gene sequences for a diverse group of bacteria in a clinical laboratory. J Clin Microbiol 44:4400–4406. doi:10.1128/JCM.01364-06. 17050811 PMC1698390

[23] Simmon KE, Mirrett S, Reller LB, Petti CA. 2008. Genotypic diversity of anaerobic isolates from bloodstream infections. J Clin Microbiol 46:1596–1601. doi:10.1128/JCM.02469-07. 18322067 PMC2395081

[24] European Committee on Antimicrobial Susceptibility Testing. 2020. Breakpoint tables for interpretation of MICs and zone diameters, pp 93–94. version 10.0. https://www.eucast.org/fileadmin/src/media/PDFs/EUCAST_files/Breakpoint-t00les/v_10.0_Breakpoint-T00les.pdf.

[25] Tamura K, Peterson D, Peterson N, Stecher G, Nei M, Kumar S. 2011. MEGA5: Molecular Evolutionary Genetics Analysis using maximum likelihood, evolutionary distance, and maximum parsimony methods. Mol Biol Evol 28:2731–2739. doi:10.1093/molbev/msr121. 21546353 PMC3203626

[26] Rambaut A. 2018. FigTree v1.4.4. https://github.com/rambaut/figtree.

[27] Johansen TEB, Botto H, Cek M, Grabe M, Tenke P, Wagenlehner FM, Naber KG. 2011. Critical review of current definitions of urinary tract infections and proposal of an EAU/ESIU classification system. Int J Antimicrob Agents 38:64–70. doi:10.1016/j.ijantimicag.2011.09.009. 22018988

